# Prevalence of SARS-CoV-2 antibodies and risk factors in the pandemic epicentre of Catalonia

**DOI:** 10.1038/s41598-022-13290-2

**Published:** 2022-06-02

**Authors:** Sandra Moreno, Queralt Miró, Ainhoa Soler, Mireia Gallego, Maria Homs, Maria José Garcia

**Affiliations:** 1 Centro de Atención Primaria de Vilanova del Camí, Institut Català de La Salut, Catalunya Central, Vilanova del Camí, Barcelona Spain; 2 Health Promotion in Rural Areas Research Group, Gerència Territorial de La Catalunya Central, Institut Català de La Salut, Carrer Pica d’Estats, 36, 08272 Sant Fruitós de Bages, Barcelona Spain

**Keywords:** Viral infection, Health care, Risk factors

## Abstract

To define the seroprevalence of antibodies against SARS-CoV-2 in the municipality of Vilanova del Camí (in the region of Conca d'Ódena, Barcelona, Spain) and to know the risk factors associated with positive seroprevalence. Cross-sectional descriptive study. The population of Vilanova del Camí had the opportunity to voluntarily attend two screenings (October and December 2020) for antibodies against the nucleocapsid protein of SARS-CoV-2 using a Rapid Diagnostic Test (RDT) (Salocor (Salofa Oy). Participants in the screening signed an informed consent form. From the 3,610 attendees at the screening, 2,170 patients were randomly selected. The relationship between antibody test results and other demographic (sex, age, morbidity index) and clinical (diagnoses, smoking and drugs) variables was analysed. The prevalence of antibodies against SARS-CoV-2 was 9.6% (95% CI of 8.4% to 10.9%) and was similar for men and women but increased with age. Among complex chronic patients, 14.3% had antibodies against SARS-CoV-2, and among patients with advanced chronic disease, 25% had antibodies against SARS-CoV-2. Age, AMG (Adjusted Morbidity Groups) index, COVID-19 diagnosis and contact with a COVID-19 case were risk factors for positive seroprevalence. A higher seroprevalence was detected in the October screening (12.16%) than in the December screening (8.38%). In the December screening, obesity was a risk factor for positive seroprevalence. This study demonstrates the high seroprevalence of antibodies against SARS-CoV-2 in the pandemic epicentre of Catalonia.

## Introduction

Severe Acute Respiratory Syndrome Coronavirus 2 (SARS-CoV-2) was identified in December 2019 as the cause of the COVID-19 disease^1^. On March 11, 2020, the World Health Organization (WHO) declared a pandemic for COVID-19^2^. The Spanish Government approved a Royal Decree (RD 463/2020 of 14 March) to establish a state of alarm to manage the health crisis. Since then, successive decrees have been passed to establish measures aimed at minimising population mobility and the spread of the virus.

In March 2020, Conca d'Ódena was considered the national pandemic epicentre in Spain, implying a strict confinement from 12th March 6th to April, 2020^3^. Conca d'Ódena is formed by the grouping of several municipalities in the province of Barcelona, in the region of Anoia: Igualada, Vilanova del Camí, Santa Margarida de Montbui and Ódena. It is estimated that 40% of health professionals in the Conca d’Ódena were COVID-19 cases or contacts during the month of March, although there are no official published data. At the beginning of the pandemic, no diagnostic tests were available and many of the possible cases were suspected cases of COVID-19 without antigenic confirmation.

Spain is one of the European countries most severely affected by the pandemic^4^. Up to March 2021, the accumulated incidence at the state level amounted to 4,967,200 cases, of which 86,621 have died, and 912,546 cases in Catalonia, of which 15,494 have died. In Anoia County from 01/03/2020 to 24/03/2021, 7,460 cases and 618 deaths due to COVID-19 were registered. Although it is recognised that the territory of Conca d'Ódena suffered severely from the impact of COVID-19 during the first wave, the percentage of the population with antibodies against SARS-CoV-2 at that time is unknown and we will most likely never know the number of real cases because diagnostic tests were not available at the beginning.

Seroprevalence studies quantify the proportion of the population that has antibodies against a pathogen. Most SARS-CoV-2 seroprevalence studies detect IgG-type antibodies^5^. Several seroprevalence studies have been published worldwide^6–10^. The Spanish study published in The Lancet, ENE-COVID^11^, analysed more than 61,000 randomly selected individuals between 27th April and 11th May, 2020 using immunochromatography and chemiluminescence tests. In this study, a seroprevalence of 5% was established in the Spanish state and a seroprevalence of 7% was established in the province of Barcelona. The study detected differences between provinces and ages, but no gender differences^11^. An Italian seroprevalence study found a higher prevalence of antibodies in women than in men^12^.

The risk factors for the disease are unknown, although some associated with positive seroprevalence have been established. The main ones are: being a contact of a positive case^13^ and being a health worker^14^. In the Dutch population, 2.7% of antibodies were found to be present and the following risk factors were detected: the Protestant religious community and the use of immunosuppressants, antivirals or antibiotics in the last month^15^.

According to the report of the ISCIII Coronavirus Scientific Analysis Group (GACC-*ISCIII)^16^, COVID-19 does not affect everyone equally. The incidence and severity of the disease is related to the presence of chronic diseases. Comorbidities could explain some of the differences according to gender and age. Possible risk factors associated with COVID-19 include: diabetes mellitus, chronic respiratory disease, chronic renal failure, cancer, immunosuppression, obesity, cardiovascular disease and smoking^16^.

The objective of this study was to define the seroprevalence of antibodies against SARS-CoV-2 in the municipality of Vilanova del Camí (Conca d'Ódena) in the first and second wave of COVID-19 in Spain, and its relationship with main demographic and clinical variables.

## Results

In this study, 2170 participants were analysed, with a median age of 47 years and of which 59% were women. Of the total number of participants, 1% were CCP and 0.2% ACD. The migrant population attending screening represented 1.6%, and 38.8% of participants had an AMG index of 2 (low risk), 22.7% had an AMG index of 3 (moderate risk) and 5.0% had an AMG index of 4 (high risk) (Table 1).

**Table 1:** Characteristics of the total population, the first screening and the second screening.

	Total (n = 2170)	1st Screening (n = 691)	2nd Screening (n = 1.479)	*p*-value
Women	1281 (59.03)	417 (60.3%)	864 (58.4%)	0.421
Age*	47.4 (20.09)	47.21 (20.98%)	48.27 (19.65)	
**Age group**				0.078
Under 20 years old	255 (11.75)	97 (14.0%)	158 (10.7%)	
20–39 years old	403 (18.57)	129 (18.7%)	274 (18.5%)	
40–59 years old	842 (38.80)	248 (35.9%)	594 (40.2%)	
60 years of age or older	670 (30.87)	217 (31.4%)	453 (30.6%)	
**Number of diagnoses**				0.216
None	1107 (51.06)	337 (48.9%)	770 (52.1%)	
1	595 (27.44)	195 (28.3%)	400 (27.0%)	
2	252 (11.62)	77 (11.2%)	175 (11.8%)	
3 or more	214 (9.87)	80 (11.6%)	134 (9.06%)	
CCP	21 (0.97)	13 (1.88%)	8 (0.54%)	**0.006**
ACD	4 (0.18)	2 (0.29%)	2 (0.14%)	0.596
Migrants	32 (1.63)	7 (1.10%)	25 (1.89%)	0.271
**AMG**				**0.105**
1	709 (33.28)	207 (30.5%)	502 (34.6%)	
2	827 (38.83)	272 (40.1%)	555 (38.2%)	
3	487 (22.86)	156 (23.0%)	331 (22.8%)	
4	107 (5.02)	43 (6.34%)	64 (4.41%)	
COVID-19 diagnosis (previous 6 m)	123 (5.67)	78 (11.3%)	45 (3.04%)	** < 0.001**
Contact COVID-19 (previous 6 m)	233 (10.74)	60 (8.68%)	173 (11.7%)	**0.042**
Cancer	387 (17.83)	143 (20.7%)	244 (16.5%)	**0.020**
Cardiopathy	35 (1.61)	15 (2.17%)	20 (1.35%)	0.220
DM	194 (8.94)	65 (9.41%)	129 (8.72%)	0.660
HTA	459 (21.15)	159 (23.0%)	300 (20.3%)	0.164
Kidney failure	76 (3.5)	27 (3.91%)	49 (3.31%)	0.564
Obesity	527 (24.29)	173 (25.0%)	354 (23.9%)	0.615
Respiratory disease	154 (7.1)	51 (7.38%)	103 (6.96%)	0.793
Smoking diagnosis	356 (16.41)	105 (15.2%)	251 (17.0%)	0.328
Prescription antibiotics	195 (8.99)	73 (10.6%)	122 (8.25%)	0.094
Prescription antivirals	9 (0.41)	3 (0.43%)	6 (0.41%)	1.000
Prescription immunosuppressants	14 (0.65)	4 (0.58%)	10 (0.68%)	1.000

The *p*-value expresses the comparison between the first and second screening population.

*The mean and standard deviation were used to describe the variable of age.

Just under half of the participants had one or more diagnoses. The diagnoses detected were obesity (24.3%), cancer (17.8%), type II diabetes (8.9%), respiratory disease (7.10%), renal failure (3.5%) and heart disease (1.6%). Of the possible risk factors described, smoking (16.4%), prescription of antibiotics (8.9%), antivirals (0.4%) and immunosuppressants (0.6%) one month prior to the test were analysed (Table 1).

A total of 5.7% of the population analysed had a diagnosis of COVID-19 in the 6 months prior to the test and 10.7% had a diagnosis of close contact with a COVID-19 case in the 6 months prior to the test (Table 1).

**Figure 1:**
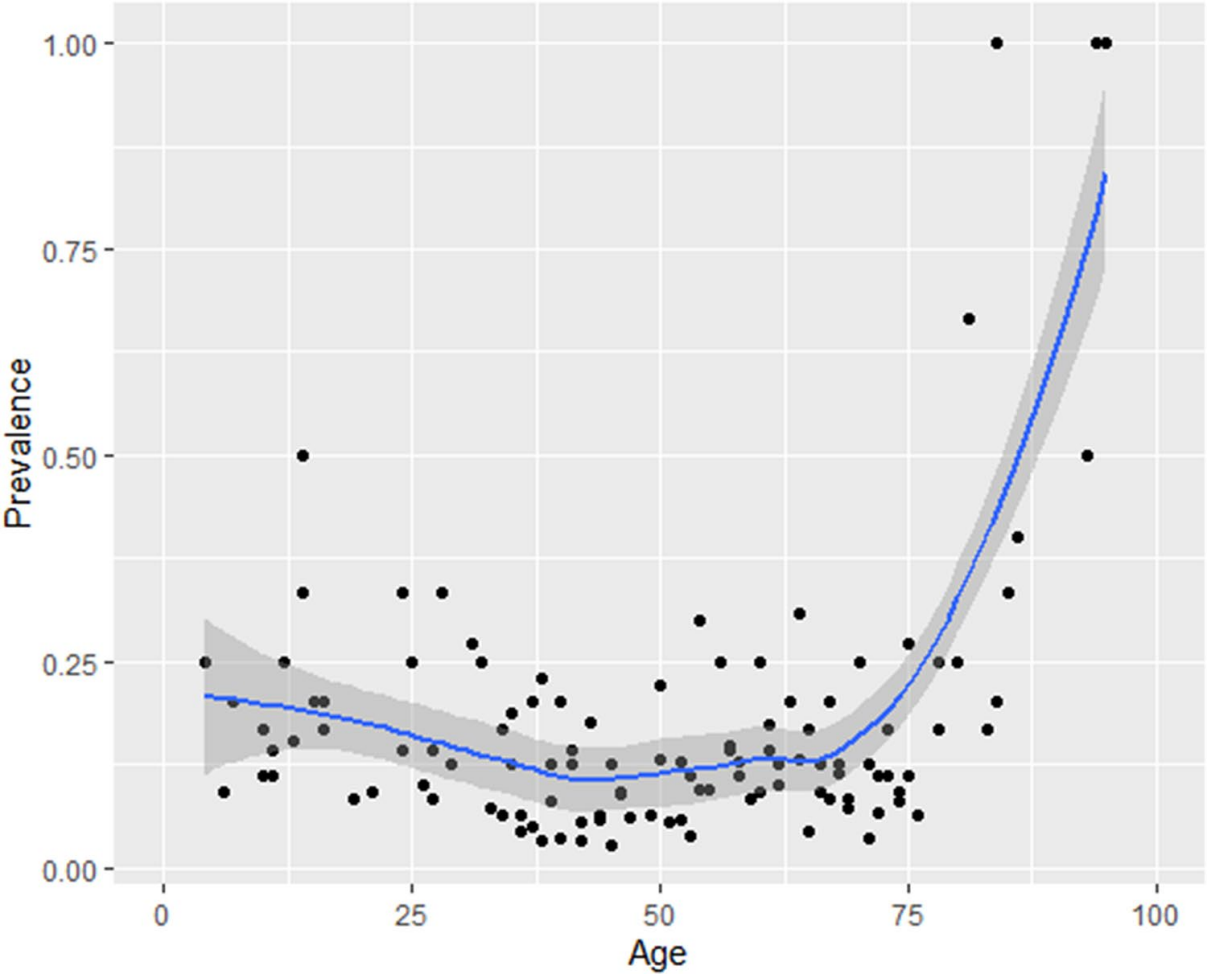
Relation between SARS-CoV-2 antibody seroprevalence and age.

The prevalence of antibodies against SARS-CoV-2 detected in the population analysed was 9.6%, with a 95% CI of 8.4% to 10.9%. By sex, the prevalence was similar between men and women: 9.2% and 10.1% respectively (*p*-value = 0.525). The seroprevalence of antibodies increased with age (Fig. 1) and this increase was significant in the older age group (*p*-value = 0.008): in participants under 20 years of age, the prevalence was 8.2%, in those aged 20–39 years 8.2%, in those aged 40–59 years 8.1% and in those over 59 years, the prevalence was 12.8% (Table 2). In CCP, 14.3% had antibodies against SARS-CoV-2 and in ACD, 25%.

**Table 2:** Prevalence and prevalence ratio of the variables analysed, in the total sample and in the first and second screening.

	Total	PR	*P*-value	1st Screening	PR	*P*-value	2nd Screening	PR	*P*-value
Total sample	208 (9.58)	(8.42; 10.89)		84 (12.16)	(9.93; 14.80)		124 (8.38)	(7.01; 9.91)	
Females	118 (9.21)	0.91 (0.70; 1.18)	0.525	49 (11.8)	0.92 (0.61; 1.38)	0.776	69 (7.99)	0.89 (0.64; 1.25)	0.576
**Age group**			**0.008**			0.705			**0.003**
Under 20	21 (8.23)	Ref		11c(11,34)	Ref		10 (6.33)	Ref	
Between 20 and 39	33 (8.19)	0.99 (0.58; 1.74)		17 (13.17)	1.16 (0.55; 2.56)		16 (5.84)	0.92 (0.42; 2.10)	
Between 40 and 59	68 (8.08)	0.98 (0.61; 1.64)		26 (10.48)	0.92 (0.47; 1.95)		42 (7.07)	1.12 (0.58; 2.36)	
60 years of age or older	86 (12.83)	1.56 (0.99; 2.57)		30 (13.82)	1.22 (0.63; 2.54)		56 (12.36)	1.95 (1.04; 4.07)	
**Diagnostics**			0.765			0.939			
None	100 (9.03)	Ref		43 (12.76)	Ref		57 (7.40)	Ref	0.421
1	59 (9.91)	1.09 (0.79; 1.51)		24 (12.31)	0.96 (0.58; 1.57)		35 (8.75)	1.18 (0.77; 1.79)	
2	25 (9.92)	1.09 (0.69; 1.67)		8 (10.39)	0.81 (0.35; 1.64)		17 (9.71)	1.31 (0.74; 2.20)	
3 or more	24 (11.21)	1.24 (0.78; 1.90)		9 (11.25)	0.88 (0.40; 1.72)		15 (11.19)	1.51 (0.82; 2.59)	
CCP	3 (14.29)	1.50 (0.52; 4.30)	0.446	2 (15.4)	1.27 (0.35; 4.63)	0.665	1 (12.5)	1.49 (0.24; 9.42)	0.504
ACD	1 (25.00)	2.62 (0.48; 14.35)	0.332	1 (50.0)	4.15 (1.02; 16.84)	0.228	0 (0.0)	-	1
**Origin**			0.129			0.605			**0.018**
Native	190 (9.85)	Ref		79 (12.5)	Ref		111 (8.55)	Ref	
Migrant	6 (18.75)	1.90 (0.91; 3.97)		0 (0.0)	-		6 (24.00)	2.81 (1.37; 5.77)	
**AMG**			**0.043**			0.435			0.095
1	52 (7.33)	Ref		19 (9.17)	Ref		33 (6.57)	Ref	
2	84 (10.16)	1.38 (0.98; 1.97)		38 (13.97)	1.52 (0.89; 2.69)		46 (8.29)	1.26 (0.81; 1.98)	
3	54 (11.09)	1.51 (1.03; 2.22)		20 (12.82)	1.39 (0.74; 2.63)		34 (10.27)	1.56 (0.97; 2.53)	
4	15 (14.02)	1.91 (1.04; 3.31)		6 (13.95)	1.52 (0.55; 3.59)		9 (14.06)	2.14 (0.96; 4.28)	
Covid Diagnostics	104 (84.55)	16.64 (13.60; 20.37)	** < 0.001**	75 (96.15)	65.49 (34.19; 125.45)	** < 0.001**	29 (64.44)	9.73 (7.27; 13.02)	** < 0.001**
Contact COVID	32 (13.73)	1.51 (1.06; 2.15)	**0.031**	7 (11.7)	0.96 (0.46; 1.98)	1	25 (14.45)	1.91 (1.27; 2.87)	**0.003**
Cancer	39 (10.08)	1.06 (0.76; 1.48)	0.789	18 (12.6)	1.05 (0.64; 1.70)	0.973	21 (8.61)	1.03 (0.66; 1.62)	0.991
Cardiopathy	4 (11.43)	1.20 (0.47; 3.04)	0.572	1 (6.67)	0.54 (0.08; 3.65)	1	3 (15.0)	1.81 (0.63; 5.21)	0.232
DM	19 (9.79)	1.02 (0.65; 1.60)	1	6 (9.23)	0.74 (0.34; 1.63)	0.576	13 (10.08)	1.23 (0.71; 2.11)	0.575
HTA	44 (9.59)	1.00 (0.73; 1.37)	1	19 (11.9)	0.98 (0.61; 1.58)	1	25 (8.33)	0.99 (0.65; 1.51)	1
Kidney failure	9 (11.84)	1.25 (0.67; 2.33)	0.629	2 (7.41)	0.60 (0.16; 2.31)	0.638	7 (14.29)	1.75 (0.86; 3.54)	0.181
Obesity	62 (11.76)	1.32 (1.00; 1.75)	0.062	22 (12.7)	1.06 (0.67; 1.67)	0.899	40 (11.30)	1.51 (1.06; 2.16)	**0.031**
Respiratory disease	13 (8.44)	0.87 (0.51; 1.49)	0.72	4 (7.84)	0.63 (0.24; 1.64)	0.449	9 (8.74)	1.05 (0.55; 2.00)	1
Smoking	22 (6.18)	0.60 (0.39; 0.92)	0.022	14 (13.3)	1.12 (0.65; 1.91)	0.811	8 (3.19)	0.34 (0.17; 0.68)	**0.002**
Antibiotics	19 (9.74)	1.02 (0.65; 1.59)	1	6 (8.22)	0.65 (0.29; 1.44)	0.368	13 (10.66)	1.30 (0.76; 2.24)	0.438
Antivirals	2 (22.22)	2.33 (0.68; 7.79)	0.212	0 (0.0)	Ref	1	2 (33.33)	4.02 (1.28; 12.64)	0.083
Immunosuppressants	1 (7.14)	0.74 (0.11; 4.94)	1	1 (25.0)	2.07 (0.37; 11.43)	0.405	0 (0.0)	-	1

* They are represented by n and percentages.

The reference category for comparison has been the No condition. No significances were detected in multivariate adjusted regressions.

The seroprevalence was significantly different according to the AMG morbidity index (*p* = 0.043), with an increase from lower to higher AMG index: in AMG of 1, the prevalence was 7.3%, in AMG of 2, 10.2%, in AMG of 3, 11.1% and in AMG of 4, the prevalence was 14.0% (Table 2). The prevalence of antibodies was also higher in cases with a diagnosis or contact diagnosis of COVID-19 (Table 2).

The seroprevalence of antibodies against SARS-CoV-2 was similar among participants with or without a diagnosis of other pathologies, such as cancer, heart disease, diabetes, arterial hypertension, renal failure, obesity or respiratory disease. There was also no difference in antibody seroprevalence between participants who took antibiotics, antivirals or immunosuppressants and those who did not. In contrast, participants who smoked had a lower prevalence of antibodies than non-smoking participants, with a PR = 0.60 (0.39, 0.92) (*p*-value = 0.022) (Table 2).

The study was conducted in two stages: a first one in October and a second one in December 2020. The period between 15th October and the end of December marked the second wave of COVID-19 cases. The first screening sample was similar to the second screening sample in terms of age, percentage of women, AMG levels and total number of diagnoses. The number of CCPs tested was significantly higher in the first screening (*p*-value = 0.006) in which there was also a higher percentage of participants who had been COVID + in the last 6 months (*p*-value < 0,001) (Table 1).

As for individual diagnoses, only the percentage of cancer showed differences, with a higher percentage in the first sample (20.7% vs. 16.5%, *p*-value = 0.020) (Table 1).

The seroprevalence of antibodies against SARS-CoV-2 was significantly higher in the October analysis, 12.16% CI95: 9.93;14.80, than in the December analysis, 8.38% CI95: 7.01;9.91 (Table 2).

In the October screening, no statistically significant associations were observed between the factors analysed and the detection of COVID-19 antibodies, except for the positive relationship between those who had experienced COVID-19 six months earlier and the positive antibody result. In contrast, in the second screening, a significant association was detected between antibody prevalence and categorised age, origin, obesity, smoking and having been COVID-19 positive or in close contact with a COVID-19 case in the last 6 months (Table 2).

## Discussion

Conca d'Ódena was considered to be ground zero of the pandemic at state level^3^. This is the first study to analyse the presence of antibodies against SARS-CoV-2 in this area. The seroprevalence of antibodies in the population analysed was 9.6%, a value higher than the seroprevalence of 7% identified in the Barcelona region, from 27th April to 11th May, 2020^11^. The results of this study might show the impact of the first wave of COVID-19 in the region. If we consider only the analysis of October, the prevalence increases to 12.16%. To date, the impact of the COVID-19 disease on a municipality in the region had not been analysed; in fact, in March 2020, COVID-19 cases were not diagnosed with tests, rather they were based merely on suspicion. Although no official mortality data per municipality are available for 2020, the written press and data from the local funeral home established an approximate increase in mortality of 386%, therefore the real seroprevalence could be much higher.

SARS-CoV-2 seroprevalence has been published in different areas of the word and in different periods^11–15,17,18^, therefore results of seroprevalence are difficult to compare. However, the meta-analysis of studies published in 2020 showed that seroprevalence in southern Europe was 4.41%^6^.This study showed that seroprevalence varied markedly among geographic regions and suggested an association of seroprevalence with income levels, human development indices, geographic latitudes and/or climate^6^.

The seroprevalence by gender was similar, as noted in some studies^12^. In contrast, a significantly higher prevalence was observed in the over-60 age group than in the younger age groups. These results could confirm the suspicion of the high impact of the first wave in older people, in fact, antibodies against SARS-CoV-2 in CCP (14.29%) and ACD (25%) were higher than within the population without CCP or ACD condition.

The AMG index of the Vilanova del Camí population is similar to that observed in the screened population. The prevalence ratio showed that a high AMG (AMG = 4) is a risk factor for having suffered from COVID-19. The AMG^16^ Index is a morbidity grouper that allows for the stratification of populations. Therefore, the higher the population morbidity, the higher the risk of having suffered from COVID-19 and therefore of having antibodies.

It has been suggested that in populations with lower socio-economic levels, the impact of COVID-19 is greater than in higher socio-economic levels^19–21^. The population of Vilanova del Camí has a high prevalence of antibodies (above the average for Barcelona and Spain) and has a low socio-economic index. Therefore, it corresponds with other studies, which consider that there are more problems in carrying out self-isolation and quarantine correctly among close contacts.

Analysis of diagnoses and seroprevalence only revealed that obesity could be a risk factor for having a higher seroprevalence of antibodies against SARS-CoV-2. Several studies have shown that obesity is a risk factor for developing the disease and for developing a more severe coronavirus disease^22,23^. It should be noted that this increased risk is for all ages. Obesity is an important public health problem. In this study, 24.3% of the screened participants were obese, a high figure, which is also related to populations with a low socio-economic level.

Other risk factors were also analysed, such as the prescription of antivirals or immunosuppressants, but very few cases of participants with an active prescription of these drugs were observed in the sample analysed.

The study was conducted in two months (October and December). The population between the first and second screening was similar (Table 1). It should be noted that, in October, a higher seroprevalence was detected than in December. These results could be explained by the fact that ’antibodies against SARS-CoV-2 decrease over time^24–26^.

Interestingly, the first screening did not detect risk factors for seroprevalence. On the contrary, in December they did observe factors previously described in the literature: age, country of origin, obesity and smoking. This difference in risk factors in the two periods of analysis could be explained by the fact that in December the behaviour of COVID-19 in Vilanova del Camí was more like other territories, unlike in October when the effects of the first wave were carried over and where the mortality rate was higher.

The main limitation of our study is the method used, as the RDT is less sensitive than ELISA, and therefore the exposure of SARS-CoV-2 in the area might be infradetected. However, in our study we detected antibodies against the nucleocapsid, which are sustained in non-severe COVID-19^27^ cases, in contrast to antibodies against the receptor-binding domain of SARS-CoV-2 spike protein, which are known to decay rapidly in patients with mild COVID-19^28^ cases.

This study demonstrates the high seroprevalence of antibodies in the pandemic epicentre of Catalonia. In October 2020, seroprevalence was 12.16% and in December 2020, 8.38%. Risk factors associated with positive seroprevalence were age, AMG index, COVID-19 diagnosis and contact diagnosis. Obesity was also a risk factor for a positive seroprevalence in the December screening. These results might show the impact of the first wave of COVID-19 in Conca d'Ódena. However, the real impact might never be known due to the initial lack of diagnostic tests, high mortality and the fact that antibodies decay over time^28^.

## Methods

The study design is observational, descriptive and cross-sectional. The reference population of the study was all the residents of the municipality of Vilanova del Camí. The study population was the resident population of Vilanova del Camí who voluntarily attended the open call screening of antibodies against the nucleocapsid protein of SARS-CoV-2, as a result of a collaboration between the Vilanova del Camí Town Council, the primary care centre of the Catalan Health Institute of Vilanova del Camí and the August Pi i Sunyer Biomedical Research Institute (IDIBAPS), and with the support of the European Institute of Innovation & Technology (EIT Health) through the Certify.health COVID-19 Rapid Response Innovation Project. The inclusion criteria for serology were being a resident in the municipality of Vilanova del Camí, being older than 1 year and accepting the informed consent. The only exclusion criterion was the presence of symptoms compatible with an active COVID-19 infection.

The municipality of Vilanova del Camí has 12,361 inhabitants, and 3610 participated in the screening from which a random sample of 2170 individuals was selected to estimate, with a confidence interval of 95% and a precision of + /− 1 percentage units, a prevalence expected to be around 7%^10^. This sample was selected from the register of participants in the primary care centre diaries, eliminating duplicate participants and expecting 5% of necessary replacements.

Samples were taken from capillary blood draws. The rapid serological test to determine the presence of antibodies against the nucleocapsid protein of SARS-CoV-2 was Salocor (Salofa Oy), with a sensitivity of 93.7% and a specificity of 99.9% to detect IgG declared by the manufacturer. The antibody result (IgG) was recorded in the corresponding variable of the Primary Care Clinical Workstation. The variables analysed in the study were obtained from the existing electronic clinical record of the participants, and were: sex, age, diagnosis of COVID-19, close contact with a COVID-19 case during the 6 months prior to the date of the test, origin of the population (native/migrant), and prescription of immunosuppressants, antivirals or antibiotics in the last month of the test. The registry of diagnoses of arterial hypertension, diabetes mellitus, chronic respiratory disease (bronchial asthma or COPD), chronic renal failure, cancer, obesity, heart disease and smoking were also analysed. In addition, the conditions of Complex Chronic Patient (CCP), of patients with Advanced Chronic Disease (ACD) and the AMG (Adjusted Morbidity Groups) index, used to elaborate the health risk strata pyramid of the general population of Catalonia, were considered^29^.

A univariate analysis of qualitative variables (absolute frequency and percentage) and quantitative variables (mean and standard deviation or median and quartiles, according to normality distribution) was performed. Bivariate studies of the dependent variables and the independent variables were also carried out. The prevalence of the total sample was calculated, and the prevalence ratio was estimated according to the potential variables in order to estimate the magnitude of association. Multivariate regressions (Generalized Linear Models (GLM) with Poisson family and robust variances) adjusted by significant independent variables from univariate analysis and potential confounding variables was also performed. Statistically significant differences were considered statistically significant with *p*-values of < 0.05 and 95% confidence intervals. All analyses were performed with version 5.3.2 of the statistical program R.

The screening had the approval of the ethics committee of Hospital Clínic of Barcelona (HCB/2020/0709) and this study has the approval of the Ethics Committee from the Foundation University Institute for Primary Health Care Research Jordi Gol i Gurina (IDIAPJGol) (21/136-PCV). All participants signed informed consent forms. This study was conducted in accordance with the latest revised ethical guidelines of the Declaration of Helsinki.
